# Recurrence Rate of Melanoma in Situ when Treated with Serial Disk Staged Excision: A Case Series

**DOI:** 10.13188/2373-1044.1000037

**Published:** 2017-02-27

**Authors:** Daniel Garcia, Robert E Eilers, S. Brian Jiang

**Affiliations:** Department of Dermatology, Dermatologic and Mohs Micrographic Surgery Center, San Diego School of Medicine, University of California

**Keywords:** Melanoma in situ (MIS), Lentigo maligna (LM), Malignant melanoma (MM), Excision, Staged excision, Skin, Dermatology

## Abstract

**Background:**

Cutaneous melanoma is one of the fastest rising cancer diagnoses in recent years. Melanoma in situ (MIS) constitutes a large proportion of all diagnosed melanomas. While surgical excision is considered the standard of therapy, the literature is not clear on which surgical technique minimizes local recurrence. A common technique is serial staged excision (SSE), in which a series of mapped excisions are made according to histopathological examination of tissue. Previously published recurrence rates for SSE ranges from 0–12%, over a range of 4.7–97 months of mean follow-up.

**Objective:**

To investigate the recurrence rate of MIS when excised using a serial disk staged excision technique with tissue marked at 12 O’clock for mapping, rush permanent processing and histologic examination, 3-suture tagging for subsequent stages, and “breadloafing” microscopic analysis. Additionally, to determine the relationship between initial lesion size and subsequent stages of excision required for clearance, and final surgical margin.

**Methods:**

Single-institution retrospective chart review of 29 biopsy confirmed MIS lesions treated with our variant of SSE. Statistical analysis via independent t-tests.

**Results:**

No recurrences were observed with mean follow-up of 31.5 months (SD 13.9), over range of 12–58 months. Mean surgical margin of 13.1 mm (SD 5.9). A trend towards larger surgical margin was seen with increasing pre-operative lesion size.

**Conclusion:**

This method of SSE for treatment of MIS is comparable in efficacy to other SSE techniques, and may offer physicians a relatively simple, efficacious, and accessible alternative to wide local excision and Mohs micrographic surgery.

## Introduction

Cutaneous melanoma is one of the fastest rising cancer diagnoses in recent years [1]. Both melanoma in Situ (MIS), an early melanoma confined to the epidermis, and invasive melanoma incidence is on the rise, making effective treatment of MIS an area of opportunity where further knowledge on the treatment outcomes of various surgical modalities could possibly reduce the burden of invasive melanoma [2]. Lentigo maligna (LM) is a subtype of MIS that has been of recent interest in the literature due to its differences in behavior and outcome compared to non-LM MIS. These differences include a tendency towards subclinical peripheral extension, and difficulty of histological diagnosis when located in sun-damaged skin. LM is considered the in-situ precursor to Lentigo maligna melanoma (LMM), an invasive form of melanoma. In the past LM and LMM were thought to be relatively benign subtypes of melanoma, however an epidemiological study has suggested that untreated Lentigo maligna has an overall lifetime risk of progressing to LMM of 2.2–4.7%, which when controlling for depth of invasion has a similar prognosis to other forms of Melanoma [3].

While non-surgical approaches such as cryotherapy, imiquimod, electrodessication and curettage, laser surgery, radiotherapy, and 5-FU are options under the care of an experienced physician, they collectively have high recurrence rates ranging from 20%–100% [4,5]. The current standard of therapy for all types of MIS is surgical excision within two to four weeks of diagnosis [6]. The National Comprehensive Cancer Network suggests a surgical margin of 5 mm and consideration of larger margins for LM [7]. However, several studies have suggested that 5 mm is a suboptimal margin, shown by clearance rates of 24%–70% at a surgical margin of 5 mm, and recurrence rates ranging from 7%–20% [8–14]. Difficulty in visualizing and differentiating cell types on the excisional margins contribute to this inability to sufficiently excise the tumor so frequently, as well as the common localization of the tumor on aesthetically challenging areas [5,9].

Several different margin control surgical techniques have been studied, including varieties of serial staged excision (SSE) and Mohs micrographic surgery (MMS). Studies suggest better margin control and lower recurrence rates with these techniques when compared to standard wide local excision (WLE) [5,9,12,15–32]. These techniques differ in surgical procedure as well as tissue examination, such as frozen versus permanent tissue processing, and “en face” versus “breadloafing” sectioning. Frozen processing allows for more rapid examination of the tissue, while permanent preparation method allows better quality of the histology slide resolution with fewer artifacts. With “en face” examination the pathologist examines sections of tissue directly facing the edge of the surgical margin, while “breadloafing” takes radial sections that allow the pathologist to examine the changes of the cells from the center of the sample to the outside edge. Comparison of these studies is hampered by differences in duration and methods of follow up [16], and no randomized clinical trials have compared the different surgical or microscopic examination techniques [4]. One Cochrane Database analysis found that there is a lack of high-quality evidence for the treatment of MIS and LM [4]. The purpose of this study is to investigate the recurrence rate of MIS when excised using a serial disk staged excision technique with tissue marked at 12 O’clock for mapping, rush permanent processing and histologic examination, 3-suture tagging for subsequent stages, and “breadloafing” microscopic analysis.

## Materials and Methods

### Data collection

We reviewed the medical records of 39 patients at the Dermatologic Surgery Unit of the University of California, San Diego in La Jolla, CA, with MIS including LM treated with SSE as described below between July 1, 2010 and September 30, 2014. The study was approved by the University of California, San Diego Institutional Review Board before records were accessed. Information acquired included: sex, Fitzpatrick skin type, age at diagnosis and excision, pathology reports, tumor location, tumor dimensions, excision and repair dimensions, number of stages required, complications, recurrences, and duration of follow-up.

Follow up was ascertained via chart review, or phone call when the patient did not continue follow-up at UCSD Dermatology for a minimum of 12 months. During phone calls patients were asked about any new lesions arising from the surgical scar, and whether they had continued receiving follow-up under an outside dermatologist.

### Surgical technique

The variation of staged excision utilized in this series is simple disk staged excision with a notch marked at 12 O’clock for the initial stage, and three-suture technique marking for subsequent stages.

**Figure 1:**
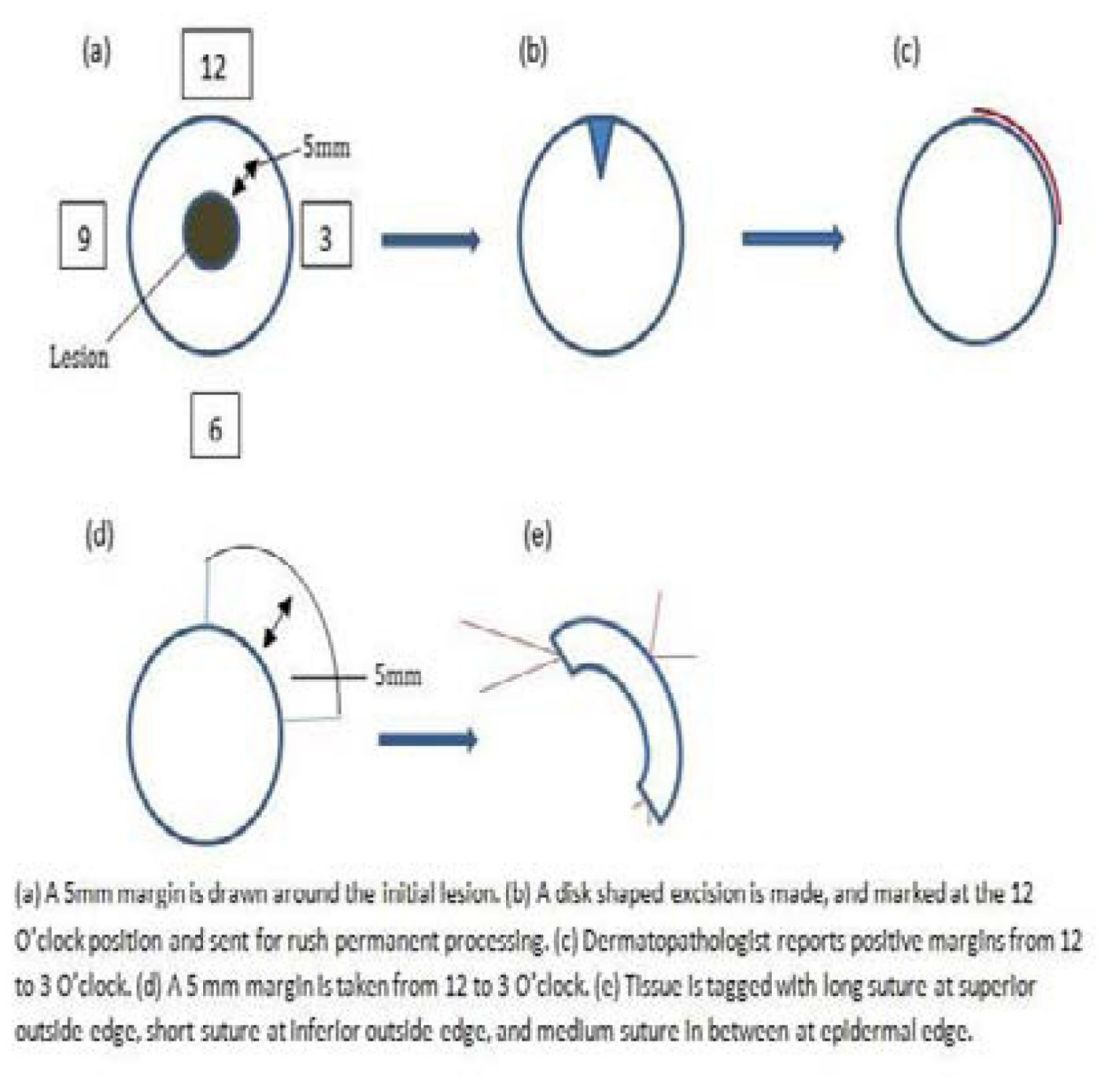
Example of serial staged excision technique investigated.

A line is drawn at approximately 5 mm around the lesion. A simple disk shaped excision is made based on this line, the tissue is marked at the 12 O’clock orientation, and sent to pathology for rush permanent section processing (Figure 1). No immunohistochemical stains are used in margin evaluation. The excised tissue is bisected and radially sectioned according to the face of a clock, and then these “hour” sections are vertically sliced, or “breadloafed”, to allow for histological examination of the tissue changes extending from the center to the edge of the sample. Within 24 hours, any presence of melanoma cells at or near the edge of the sample is marked according to the clock map, and the surgeon is informed. In between stages the wound is left open with a simple dressing in place. Subsequent stages take place within the next 2–3 days, or closure if one stage was sufficient. The next stage is mapped according to the pathologist report, and a further 5 mm excision is made conforming to this mapping. All stages beyond the first stage are marked by a long suture at the superior outside edge, small suture at the inferior outside edge, and a medium suture in between at the epidermal edge (Figure 1). The tissue is again sent off for rush permanent processing, “breadloafing”, and pathologist examination. This process is repeated for as many stages as required, until the surgeon is informed that the tissue margins are clear of melanoma cells. The resulting defect is subsequently repaired appropriately.

The decision to include the initial lesion in the first stage, as opposed to leaving until last as in some other SSE techniques such as the Johnson Square procedure [18], was made in order to more quickly identify those lesions upstaged to invasive melanomas that would be better suited for more aggressive treatment.

### Histopathologic definition

All lesions were confirmed as MIS or MIS, subtype LM by histopathological examination by a board-certified dermatopathologist. For the majority of the lesions, the biopsies were obtained and examined by our institution’s dermatopathologists. When patients were referred by outside dermatologists, biopsies were once again reviewed by our institution’s dermatopathologists, and outside pathology reports were obtained.

### Statistical analysis

Our primary outcome variable was recurrence of MIS at the original surgical site. Secondary outcomes included the number of stages required for histological clearance as well as the surgical margins required. Continuous data were analyzed via t-test and described as means with standard deviation, and medians as appropriate. The relationship between initial lesion size and margins, and initial lesion size and number of stages, was analyzed by independent t-tests. Sizes of lesions and defects were recorded by the surgeons as two orthogonal dimensions, the X and Y axis. The smaller orthogonal length of the lesion was designated preoperative size 1, and the longer as preoperative size 2. Likewise, postoperative defect sizes were recorded as postoperative size 1 and 2, and the surgical margin was defined as the largest measurement by taking the differences between postoperative sizes 1 and 2 and preoperative sizes 1 and 2, respectively.

## Results

Over the period from July 1, 2010 to September 30, 2014, 39 patients underwent staged excision for MIS. Of these 39, five subjects were dropped from analysis due to insufficient follow-up defined as less than twelve months. Another 5 subjects were dropped due to post-operative pathology revealing invasive melanoma, requiring a change to a more aggressive treatment. A total of eight subjects were contacted by phone to determine recurrence. Of these eight subjects, three had not seen a dermatologist since the last encounter in our clinic, and thus recurrence was determined by subjective report of any dark colored lesions overlying the surgical site scar. None of the subjects contacted by phone reported a recurrence or potential recurrence.

### Demographics and lesion characteristics

The final make up of our subjects was 62% male, and 38% female. The mean age at excision was 72.2 (SD 11.4) years, and the median age at excision was 72 years (range 51–93). The mean follow-up was 31.5 (SD 13.9) months, and the median follow-up was 30 months (range 12–58). The most common regions for the primary lesion were the head and neck, at 93.1% (27/29), with trunk and extremities making up the remaining 6.8% (2/29). The most common specific sites of lesion were the cheeks, with 44.8% (13/29) of total cases, followed by the temple at 13.8% (4/29), and the eyelid at 10.3% (3/29). MIS, subtype LM made up 27.6% (8/29) of all lesions, while non-LM MIS made up 72.4% (21/29).

The mean lesion size pre-operatively was 13.6 mm (SD 12.1) by 13.4 mm (SD 9.3), while the mean largest final surgical margin was 13.1 mm (SD 5.9). About 45% (13/29) of the lesions would have required larger than 5 mm margins for histological clearance. The margin required to clear 93% (27/29) of lesions was 11.9 mm, and the margin required to clear 97% (28/29) of lesions was 12.3 mm.

For all lesions, the mean number of stages required to histologically clear the tumor was 1.53 (SD 0.61). About half of the lesions (16/34, 47%) required more than one stage. The most stages required were three, for only 2 of the lesions.

We investigated the relationship of initial lesion diameter to subsequent number of stages required for clearance, as well as to final surgical margin. A trend towards larger surgical margins was seen with larger pre-operative size (Table 2). There was no clear relationship seen between pre-operative size and the number of stages required, as results were statistically insignificant (Table 3). There were no recurrences in our series of patients, comparable to other published SSE recurrence rates for MIS (Table 4).

## Discussion

In our series of patients treated with serial disk staged excision combined with mapping and “breadloafing” analysis, we had zero recurrences out of 29 subjects. This recurrence rate of 0% falls into the previously reported range for serial staged excision techniques of 0–12% [14–31]. Our mean duration of follow up of 31.5 months was likewise in the mean range of reported duration of 4.7–96 months.

While other SSE studies have found a positive relationship between initial lesion size and the margin necessary for clearance and number of stages required [19,33], our study demonstrated mixed results with statistical significance trending only for the final surgical margin. This is likely due to our study being underpowered with insufficient lesions in our analysis.

We believe our method of SSE may offer advantages over WLE for the treatment of MIS. Compared to WLE, our method of SSE offers increased rates of clearance and decreased rate of recurrence. As demonstrated in previous studies, margins of 5–6 mm as used in WLE are historically inadequate, leading to clearance rates of 0–89% and recurrence rates of 0–20% [10,13,14,34,35]. The inadequacy of 5 mm margins may be of additional significance when considering the treatment of the MIS, subtype LM, as studies have suggested that this subtype may have unpredictable sub clinical spread in the form of atypical junctional melanocytes in the deep adnexal structures with significant horizontal growth [5,36,37].

In 2008 the NCCN updated their recommendations for LM, suggesting margins of 0.5–1.0 cm and more thorough histologic analysis [7]. In our series, we found that 45% (13/29) of the lesions would have required margins larger than 5 mm for clearance. This is similar to other published results using SSE ranging from 22–58% of lesions requiring over 5 mm of margin for clearance [13,17,21,38]. Similarly, Kunishige et al. reported a series of 1072 patients with 1120 MIS lesions treated with MMS, which required 9 mm margins to clear 98.9% of lesions [10]. Our mean surgical margin was larger at 13.1 mm, although our sample size was much smaller than Kunishige et al.’s and thus more vulnerable to variation. Our results support the notion that margins larger than 5 mm are warranted in the surgical treatment of MIS on the head and neck area and are likely closer to 1.0 cm than to 5 mm.

The comparison with MMS is more complex, with MMS offering a faster procedure and 100% margin control with “en face” examination, while SSE may potentially offer more accurate histological evaluation given the better quality of permanent sections vs. frozen sections, as well as lower costs. Our SSE technique might also be more accessible to a wider audience of surgeons and specialties as it is relatively less technically complicated and does not require a Mohs laboratory in the office.

Frozen sections offer the advantage of being more time-conserving compared to permanent sections, however studies have associated frozen sections with artifactual changes such as lack of melanocyte cytoplasmic vacuolization and fixation artifacts such as bubbles, tissue folding, and chatter [12,21]. When compared to frozen sections, permanent sections have less artifactual and fixational defects, and display a characteristic morphology of atypical melanocytes as clear cells with hyperchromatic nuclei [39]. Several immunohistochemical stains have been employed with frozen sectioning in order to increase the ability to identify atypical melanocytes, including HMB-45, MEL-5, MITF, and MART-1. Despite these markers the question remains of whether “en face” examination is adequate to clearly identify the clearance of margins [39]. The choice to use vertical “breadloafing” sectioning was made to allow for examination of the evolution of cell changes as they progress to the margins, especially important when considering the difficulties in interpreting LM in the background of sun-damaged skin [40,41]. Thus, one can expect that MMS offers greater sensitivity due to the increased margin control, however it likely exhibits decreased specificity when compared to permanent section “breadloafing” techniques. Recurrence rates are somewhat comparable between SSE and MMS, with MMS demonstrating recurrence rates of 0–33%, compared to 0–12% for SSE [9]. Of note however, the recurrence rates of MMS drops to 0–6.25% when Walling et al.’s results are excluded [9,15]. Another possible tradeoff between SSE and MMS may be healthy tissue preservation, as MMS takes smaller size stages, usually 2–3 mm, however studies have demonstrated conflicting results. Zitelli et al. showed in their series that MMS spared an average of 1.8 cm of defect diameter compared to SSE, however Walling et al. found no significant difference in final defect size [15,42]. These differences can be partly attributed to surgeon preference when deciding the surgical margins (2–5 mm) for subsequent stages.

Our study had several limitations, including being a single institution study, being a retrospective chart review, lack of a comparison group as the vast majority of MIS lesions were treated with our SSE technique, and being underpowered due to too few lesions to draw from in our study timeline. In addition, accessing the surgical notes retrospectively adds the possibility of misrepresenting the measured lesion and defect dimensions. A limitation in interpretation of results is the variety of methods used to calculate surgical margins in the studies we found in our literature review, making comparison difficult. Despite these limitations we believe our results demonstrate that our method of SSE for treatment of MIS including LM is a viable and successful approach that is comparable in efficacy to other SSE techniques, and may offer physicians a relatively simple, efficacious, and accessible alternative to WLE and MMS. For patients, our SSE technique may offer the advantage of decreased recurrence when compared to standard WLE, and may offer decreased costs when compared to MMS.

## Figures and Tables

**Table 1:** Patient demographics and lesion characteristics.

Serial Staged Excision for Melanoma *in situ*	
Sex	Male 18/29 (64.7%), Female 11/29 (35.3%)
Age at Excision	Mean 72.2 years (SD 11.5)
	Median 72 years (range 51–93)
Fitzpatrick Skin Type	Type 1: 2/29 (6.8%)
	Type 2: 26/29 (89.7%)
	Type 3: 1/29 (3.4%)
Lesion Location	Head and Neck: 27/29 (93.1%)
	Trunk and Extremities: 2/29 (6.8%)
Preoperative Size 1	Mean 13.6 mm (SD 12.1)
Preoperative Size 2	Mean 13.4 mm (SD 9.3)
Final Surgical Margin	Mean 13.1 mm (SD 5.9)
Stages Needed for Histologically Clear Margins	Mean 1.48 stages (range 1–3)
Follow-up Duration	Mean 31.5 months (SD 13.9)
	Median 30 months (range 12–58)
Complications	3/29 (10.3%); hematoma ×1, post-op bleeding ×2
Recurrences	0/29 (0%)

**Table 2:** Relationship of lesion size to number of stages required for clearance.

Largest Diameter of Lesion	Number	Mean Surgical Margin	P-value
≤5 mm	2/29	8.5 mm	0.1
≤10 mm	10/29	11.1 mm	0.13
≤20 mm	22/29	13.7 mm	0.2
≤30 mm	25/29	13.8 mm	<0.01
≤40 mm	26/29	13.7 mm	0.01
≤50 mm	27/29	13.4 mm	0.04

**Table 3:** Relationship of lesion size to number of stages required for clearance.

Largest Diameter of Lesion	Number	Mean Number of Stages	P-value
≤5 mm	2/29	1	0.27
≤10 mm	10/29	1.3	0.22
≤20 mm	22/29	1.5	0.83
≤30 mm	25/29	1.56	0.1
≤40 mm	26/29	1.54	0.17
≤50 mm	27/29	1.52	0.27

**Table 4:** Literature review of serial staged excision for melanoma *in situ.*

Staged Excision Studies	Recurrence	Time to Recurrence	Follow-up Duration
Walling et al. [15]	3/41 (7.3%)	24 ± 13 months	96 ± 43.6
			(range 60–240 months)
Bub et al. [16]	2/55 (3.6%)	Not reported	57 months
			(range 9–139 months)
Huilgol et al. [17]	2/125 (1.6%)	12, 40 months	38 ± 25
Johnson et al. [18]	0/35 (0%)	NA	Not reported
Hill and Gramp. [19]	1/38 (2.6%)	10 months	25 months (range 10–48 months)
Anderson et al. [20]	1/150 (0.67%)	<5 years, not defined	Not reported
Agarwal-Antal et al. [21]	0/93 (0%)	Not reported	Not reported
			“4 years after first patient”
Malhotra et al. [22]	4/109 (3.7%)	12–40 months	32±26 months
Mahoney et al. [23]	0/11 (0%)	NA	4.7 months (range 1–13 months)
Jejurikar et al. [24]	0/51 (0%)	NA	31 months (range 15–45 months)
Bosbous et al. [25]	1/59 (1.7%)	Not reported	2.2 years (range 0–10.2 years)
Lee and Ryman [26]	3/31 (9.7%)	Mean 4 years	42 months
Joyce et al. [27]	9/410 (2.2%)	Mean 29.6 months (range 8–47 months)	23 months (range 1–65 months)
de Vries et al. [28]	4/100 (4%)	37–77 months	Mean 60 months
Patel et al. [29]	1/21(4.76%)	2 years	Not reported
Lawrence et al. [30]	4/56 (12%)	Mean 4.4 years (range 0.6–7.4 years)	Minimum 5 years (range 5–9.6 years)
Gaudy-Marqueste et al. [31]	0/20 (0%)	48 months	Mean 25.36 months (range 0–72 months)
Möller et al. [32]	0/49 (0%)	NA	Median 14 months (range 1–36 months)
Garcia and Jiang	0/29 (0%)	NA	Mean 31.5 months (range 12–58 months)
